# Supplementary material for: Elephant seal dive behaviour responds consistently to changes in foraging success regardless of sex or ocean habitat
Source: PeerJ. 2025 Dec 17;13:e20378. doi: 10.7717/peerj.20378 (PMC12717847; doi:10.7717/peerj.20378)
Supplement: Supplemental Information 1 [file peerj-13-20378-s001.docx]

Supplementary material: Elephant seal dive behaviour responds consistently to changes in foraging success regardless of sex or ocean habitat

David B. Green^1,2^, Sophie Bestley^2,3,1^, Clive R. McMahon^4,1,2,5^, Mary-Anne Lea^2,1^, Robert G. Harcourt^5^, Christophe Guinet^6^ and Mark A. Hindell^2,1^

*^1^Australian Centre for Excellence in Antarctic Science (ACEAS), University of Tasmania, Hobart, TAS, Australia*

*^2^Institute for Marine and Antarctic Studies, University of Tasmania, Hobart, TAS, Australia*

*^3^Australian Antarctic Program Partnership, University of Tasmania, Hobart, TAS, Australia*

*^4^IMOS Animal Tagging, Sydney Institute of Marine Science, Mosman, 2088, NSW, Australia*

*^5^School of Natural Sciences, Macquarie University, North Ryde, NSW, Australia*

*^6^Centre d’Etudes Biologiques de Chizé, CNRS, 79360, Villiers en Bois, France*

**Abstract**

Understanding how air-breathing diving animals moderate their dive behaviour when foraging successfully is foundational in the study of their foraging ecology. Yet, this fundamental relationship remains unresolved with previous research pointing to inconsistent relationships, differing nominally according to sex, habitat type and scale. Empirically testing the relationships between dive effort responses and foraging success is further hampered because of challenges obtaining concurrent measures of behavioural responses and foraging success at sea. We compiled a multi-decadal dive dataset from 609 southern elephant seals, including their dive responses (transit rate, and relative dive and surface recovery duration) and buoyancy – changes in which provide an indirect measure of body condition change and foraging success. Using this dataset, we tested how seal dive behaviour alters when foraging remotely at sea. We found that as foraging success increased, seals increased transit (ascent, descent) rates and decreased relative dive durations for a given depth, with no response in surface recovery. Our results were consistent across sexes and foraging habitats, and account for the general effects of buoyancy on dive behaviour. The homogeneity of these findings suggests that there is a general functional response in which elephant seals perform, on average, shorter, steeper dives during periods of successful foraging. Importantly, we can align these results with predictions from the marginal value theorem (MVT), that a forager should remain in a patch only until gains drop below the neighbourhood mean. Our findings have broad-based implications for how ecologists interpret dive responses of wild marine animals, demonstrating the value of seeking independent in situ information on foraging success.

- 1. **Main text GAMM model outputs and visualisations**

Table S1: Results from GAMM model fits reported in the main text between dive behaviour metrics, foraging success and drift rate. “Sig.” denotes p-value significance at the following levels: “***” = p < 0.001, “**” = p < 0.01, “*” = p < 0.05, “.” = p < 0.1, “ns” = p > 0.1.

|  |  |  |  | **Dive metric linear term results** | | | | **Drift rate smooth results** | | |  |
| --- | --- | --- | --- | --- | --- | --- | --- | --- | --- | --- | --- |
| **Dive metric** | **Sex** | **Habitat** | **Window (days)** | **Coeff.** | **Std. Err.** | **t value** | **Sig.** | **EDF** | **F stat.** | **Sig.** | **R^2^ _adjusted_** |
| Ascent rate | Female | Shelf | 1 | 0.33 | 0.16 | 2.02 | * | 4.44 | 55.06 | *** | 0.14 |
| Ascent rate | Female | Shelf | 2 | 0.37 | 0.07 | 5.70 | *** | 2.96 | 38.63 | *** | 0.12 |
| Ascent rate | Female | Shelf | 3 | 0.22 | 0.05 | 4.12 | *** | 1.00 | 26.83 | *** | 0.08 |
| Ascent rate | Female | Shelf | 7 | 0.16 | 0.06 | 2.86 | ** | 1.00 | 7.35 | ** | 0.06 |
| Ascent rate | Female | Shelf | 10 | 0.09 | 0.05 | 1.86 | . | 1.00 | 2.94 | . | 0.04 |
| Ascent rate | Female | Oceanic | 1 | 0.33 | 0.05 | 6.16 | *** | 6.50 | 210.83 | *** | 0.18 |
| Ascent rate | Female | Oceanic | 2 | 0.22 | 0.03 | 7.69 | *** | 6.55 | 74.95 | *** | 0.18 |
| Ascent rate | Female | Oceanic | 3 | 0.10 | 0.02 | 5.06 | *** | 5.54 | 19.57 | *** | 0.11 |
| Ascent rate | Female | Oceanic | 7 | 0.05 | 0.02 | 2.50 | * | 4.20 | 4.80 | *** | 0.04 |
| Ascent rate | Female | Oceanic | 10 | 0.04 | 0.02 | 2.85 | ** | 2.44 | 11.16 | *** | 0.04 |
| Ascent rate | Male | Shelf | 1 | 0.48 | 0.07 | 6.83 | *** | 6.56 | 81.07 | *** | 0.08 |
| Ascent rate | Male | Shelf | 2 | 0.35 | 0.04 | 8.17 | *** | 5.37 | 55.70 | *** | 0.09 |
| Ascent rate | Male | Shelf | 3 | 0.22 | 0.03 | 7.42 | *** | 2.71 | 36.30 | *** | 0.06 |
| Ascent rate | Male | Shelf | 7 | 0.09 | 0.02 | 5.25 | *** | 4.55 | 5.07 | *** | 0.02 |
| Ascent rate | Male | Shelf | 10 | 0.07 | 0.02 | 4.67 | *** | 3.66 | 2.93 | * | 0.01 |
| Ascent rate | Male | Oceanic | 1 | 0.32 | 0.07 | 4.38 | *** | 4.40 | 110.34 | *** | 0.16 |
| Ascent rate | Male | Oceanic | 2 | 0.26 | 0.04 | 6.76 | *** | 4.06 | 60.74 | *** | 0.17 |
| Ascent rate | Male | Oceanic | 3 | 0.18 | 0.03 | 5.78 | *** | 3.40 | 30.69 | *** | 0.12 |
| Ascent rate | Male | Oceanic | 7 | 0.11 | 0.02 | 5.21 | *** | 3.71 | 15.25 | *** | 0.07 |
| Ascent rate | Male | Oceanic | 10 | 0.03 | 0.02 | 1.88 | . | 1.00 | 15.47 | *** | 0.04 |
| Descent rate | Female | Shelf | 1 | 0.56 | 0.20 | 2.82 | ** | 5.20 | 105.06 | *** | 0.49 |
| Descent rate | Female | Shelf | 2 | 0.33 | 0.08 | 3.94 | *** | 4.61 | 44.79 | *** | 0.39 |
| Descent rate | Female | Shelf | 3 | 0.21 | 0.07 | 3.09 | ** | 3.12 | 15.77 | *** | 0.25 |
| Descent rate | Female | Shelf | 7 | 0.17 | 0.07 | 2.39 | * | 2.48 | 5.87 | ** | 0.14 |
| Descent rate | Female | Shelf | 10 | 0.13 | 0.06 | 2.06 | * | 1.00 | 13.07 | *** | 0.11 |
| Descent rate | Female | Oceanic | 1 | 0.39 | 0.08 | 5.05 | *** | 7.76 | 271.29 | *** | 0.43 |
| Descent rate | Female | Oceanic | 2 | 0.33 | 0.05 | 6.29 | *** | 7.66 | 78.01 | *** | 0.33 |
| Descent rate | Female | Oceanic | 3 | 0.09 | 0.03 | 2.80 | ** | 6.89 | 13.61 | *** | 0.15 |
| Descent rate | Female | Oceanic | 7 | 0.00 | 0.02 | -0.19 | ns | 7.68 | 7.00 | *** | 0.03 |
| Descent rate | Female | Oceanic | 10 | 0.01 | 0.01 | 0.46 | ns | 7.98 | 11.71 | *** | 0.07 |
| Descent rate | Male | Shelf | 1 | 0.63 | 0.11 | 5.69 | *** | 7.08 | 120.12 | *** | 0.22 |
| Descent rate | Male | Shelf | 2 | 0.29 | 0.06 | 4.55 | *** | 6.64 | 45.29 | *** | 0.17 |
| Descent rate | Male | Shelf | 3 | 0.16 | 0.04 | 4.32 | *** | 2.90 | 28.16 | *** | 0.10 |
| Descent rate | Male | Shelf | 7 | 0.15 | 0.03 | 4.86 | *** | 1.00 | 15.03 | *** | 0.03 |
| Descent rate | Male | Shelf | 10 | 0.11 | 0.02 | 5.23 | *** | 7.15 | 12.37 | *** | 0.04 |
| Descent rate | Male | Oceanic | 1 | 0.38 | 0.11 | 3.57 | *** | 5.79 | 76.32 | *** | 0.25 |
| Descent rate | Male | Oceanic | 2 | 0.29 | 0.05 | 5.34 | *** | 5.74 | 39.45 | *** | 0.23 |
| Descent rate | Male | Oceanic | 3 | 0.16 | 0.04 | 3.90 | *** | 4.76 | 17.89 | *** | 0.15 |
| Descent rate | Male | Oceanic | 7 | 0.13 | 0.03 | 4.71 | *** | 4.35 | 10.79 | *** | 0.09 |
| Descent rate | Male | Oceanic | 10 | 0.16 | 0.04 | 4.46 | *** | 7.92 | 11.28 | *** | 0.10 |
| Dive residual | Female | Shelf | 1 | -2.14 | 0.39 | -5.42 | *** | 5.70 | 58.83 | *** | 0.35 |
| Dive residual | Female | Shelf | 2 | -0.98 | 0.20 | -4.88 | *** | 5.99 | 14.30 | *** | 0.22 |
| Dive residual | Female | Shelf | 3 | -0.37 | 0.18 | -2.03 | * | 4.78 | 7.86 | *** | 0.16 |
| Dive residual | Female | Shelf | 7 | -0.60 | 0.12 | -5.21 | *** | 6.12 | 12.28 | *** | 0.19 |
| Dive residual | Female | Shelf | 10 | -0.75 | 0.16 | -4.82 | *** | 5.21 | 14.32 | *** | 0.18 |
| Dive residual | Female | Oceanic | 1 | -2.33 | 0.17 | -13.75 | *** | 7.81 | 353.82 | *** | 0.43 |
| Dive residual | Female | Oceanic | 2 | -0.64 | 0.08 | -7.87 | *** | 6.74 | 27.01 | *** | 0.24 |
| Dive residual | Female | Oceanic | 3 | -0.32 | 0.06 | -5.32 | *** | 5.86 | 10.86 | *** | 0.13 |
| Dive residual | Female | Oceanic | 7 | -0.25 | 0.04 | -7.03 | *** | 6.06 | 19.43 | *** | 0.13 |
| Dive residual | Female | Oceanic | 10 | -0.39 | 0.03 | -11.64 | *** | 6.98 | 43.05 | *** | 0.17 |
| Dive residual | Male | Shelf | 1 | -1.68 | 0.20 | -8.28 | *** | 6.22 | 99.11 | *** | 0.25 |
| Dive residual | Male | Shelf | 2 | -1.00 | 0.11 | -9.06 | *** | 5.90 | 48.82 | *** | 0.20 |
| Dive residual | Male | Shelf | 3 | -0.67 | 0.08 | -8.15 | *** | 4.98 | 34.17 | *** | 0.15 |
| Dive residual | Male | Shelf | 7 | -0.54 | 0.06 | -9.06 | *** | 4.33 | 34.02 | *** | 0.11 |
| Dive residual | Male | Shelf | 10 | -0.56 | 0.06 | -9.38 | *** | 4.37 | 44.44 | *** | 0.11 |
| Dive residual | Male | Oceanic | 1 | -2.38 | 0.32 | -7.50 | *** | 5.88 | 108.71 | *** | 0.38 |
| Dive residual | Male | Oceanic | 2 | -1.21 | 0.13 | -9.50 | *** | 7.28 | 43.37 | *** | 0.32 |
| Dive residual | Male | Oceanic | 3 | -0.81 | 0.09 | -9.34 | *** | 7.09 | 27.48 | *** | 0.25 |
| Dive residual | Male | Oceanic | 7 | -0.50 | 0.08 | -5.89 | *** | 6.12 | 24.74 | *** | 0.19 |
| Dive residual | Male | Oceanic | 10 | -0.37 | 0.05 | -6.98 | *** | 6.52 | 25.64 | *** | 0.16 |
| Surface residual | Female | Shelf | 1 | 0.13 | 0.13 | 0.99 | ns | 4.36 | 5.28 | *** | 0.03 |
| Surface residual | Female | Shelf | 2 | -0.11 | 0.06 | -1.71 | . | 2.77 | 7.18 | *** | 0.04 |
| Surface residual | Female | Shelf | 3 | -0.02 | 0.06 | -0.39 | ns | 2.20 | 6.41 | ** | 0.03 |
| Surface residual | Female | Shelf | 7 | -0.02 | 0.05 | -0.43 | ns | 2.15 | 4.83 | ** | 0.03 |
| Surface residual | Female | Shelf | 10 | -0.02 | 0.05 | -0.46 | ns | 1.00 | 2.83 | . | 0.01 |
| Surface residual | Female | Oceanic | 1 | 0.02 | 0.04 | 0.50 | ns | 7.00 | 24.83 | *** | 0.05 |
| Surface residual | Female | Oceanic | 2 | -0.01 | 0.02 | -0.69 | ns | 4.39 | 20.16 | *** | 0.05 |
| Surface residual | Female | Oceanic | 3 | -0.03 | 0.01 | -2.11 | * | 4.28 | 11.67 | *** | 0.04 |
| Surface residual | Female | Oceanic | 7 | 0.02 | 0.01 | 1.52 | ns | 5.33 | 6.32 | *** | 0.02 |
| Surface residual | Female | Oceanic | 10 | -0.02 | 0.01 | -2.08 | * | 3.58 | 5.15 | *** | 0.02 |
| Surface residual | Male | Shelf | 1 | 0.05 | 0.06 | 0.75 | ns | 6.46 | 14.48 | *** | -0.01 |
| Surface residual | Male | Shelf | 2 | 0.08 | 0.03 | 2.35 | * | 4.91 | 5.86 | *** | 0.00 |
| Surface residual | Male | Shelf | 3 | 0.03 | 0.02 | 1.53 | ns | 3.97 | 2.33 | . | 0.00 |
| Surface residual | Male | Shelf | 7 | 0.02 | 0.01 | 1.61 | ns | 2.87 | 3.55 | * | 0.01 |
| Surface residual | Male | Shelf | 10 | -0.01 | 0.01 | -0.93 | ns | 3.90 | 9.29 | *** | 0.01 |
| Surface residual | Male | Oceanic | 1 | 0.10 | 0.07 | 1.38 | ns | 6.05 | 9.32 | *** | 0.01 |
| Surface residual | Male | Oceanic | 2 | 0.09 | 0.04 | 2.44 | * | 4.61 | 3.04 | * | 0.01 |
| Surface residual | Male | Oceanic | 3 | 0.02 | 0.04 | 0.57 | ns | 4.65 | 2.64 | * | 0.01 |
| Surface residual | Male | Oceanic | 7 | 0.01 | 0.02 | 0.62 | ns | 3.00 | 2.73 | * | 0.01 |
| Surface residual | Male | Oceanic | 10 | 0.04 | 0.01 | 2.93 | ** | 5.93 | 5.03 | *** | 0.01 |


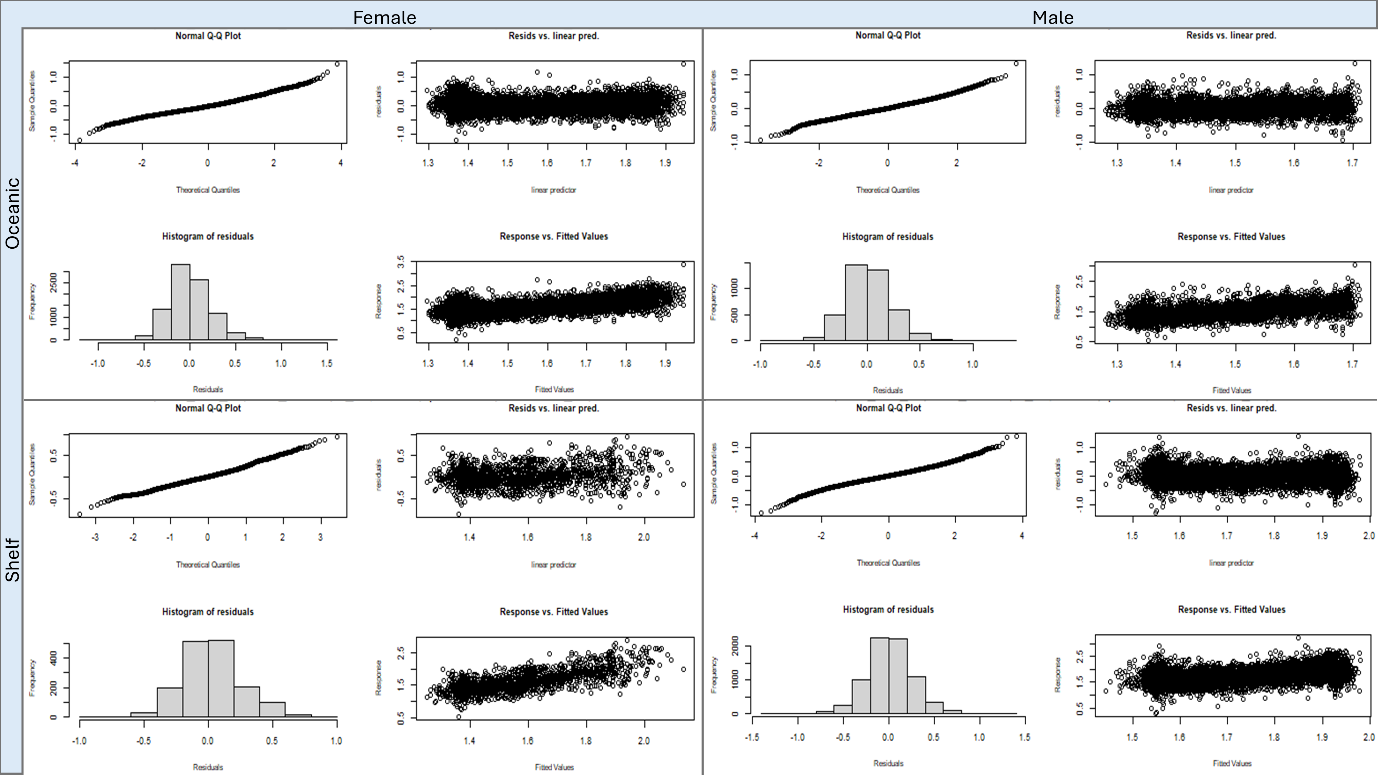


Fig. S1. Diagnostic plots from a call to “gam.check” (package mgcv; Wood 2017) for each of the GAMMs with descent rate as the response, across the 1-day temporal window. Note the clear relationship between the response and fitted values, while histograms and plots of residuals against the linear predictor show that model residuals are centred on zero, with a uniform spread around the mean.


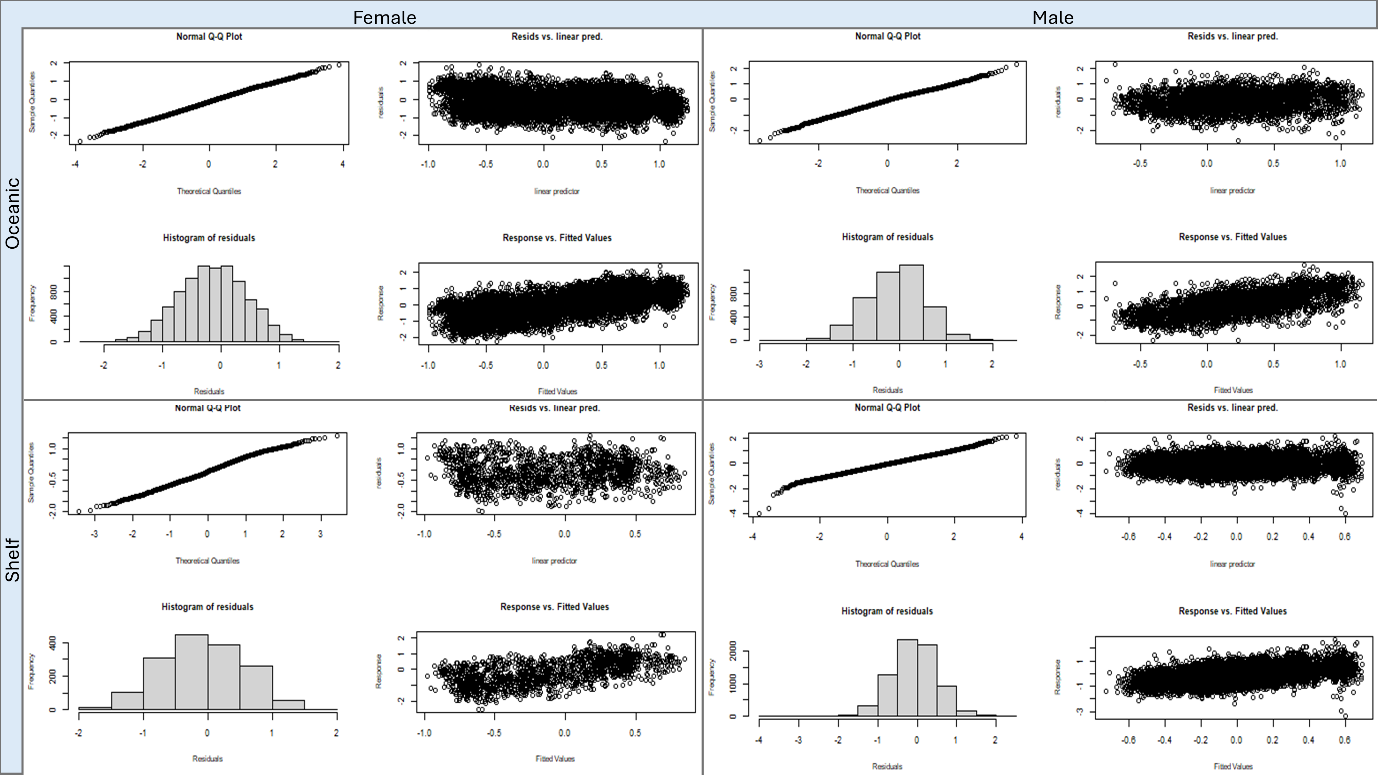


Fig. S2. Diagnostic plots from a call to “gam.check” (package mgcv; Wood 2017) for each of the GAMMs with dive residual as the response, across the 1-day temporal window. Note the clear relationship between the response and fitted values, while histograms and plots of residuals against the linear predictor show that model residuals are centred on zero, with a uniform spread around the mean.


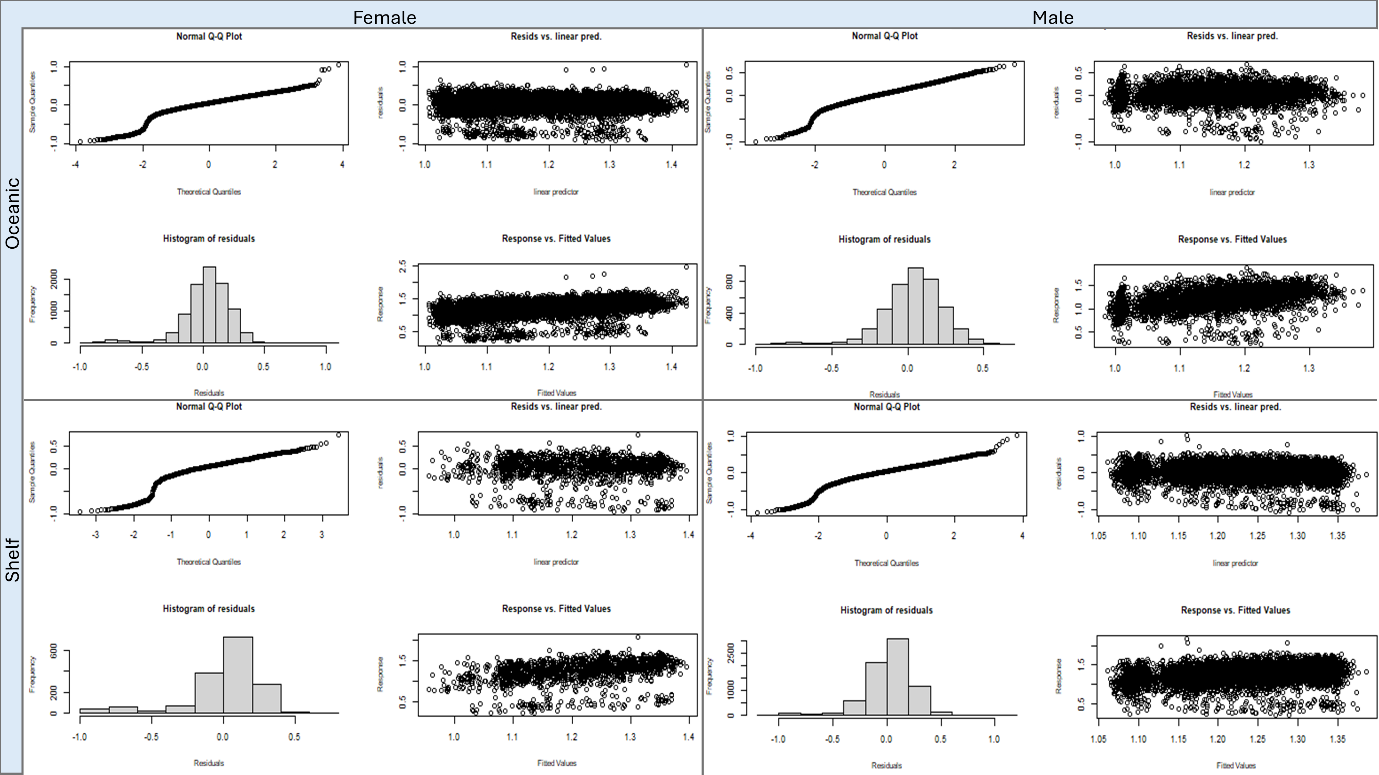


Fig. S3. Diagnostic plots from a call to “gam.check” (package mgcv; Wood 2017) for each of the GAMMs with ascent rate as the response, across the 1-day temporal window. Note the clear relationship between the response and fitted values, while histograms and plots of residuals against the linear predictor show that model residuals are centred on zero, with a generally uniform spread around the mean.


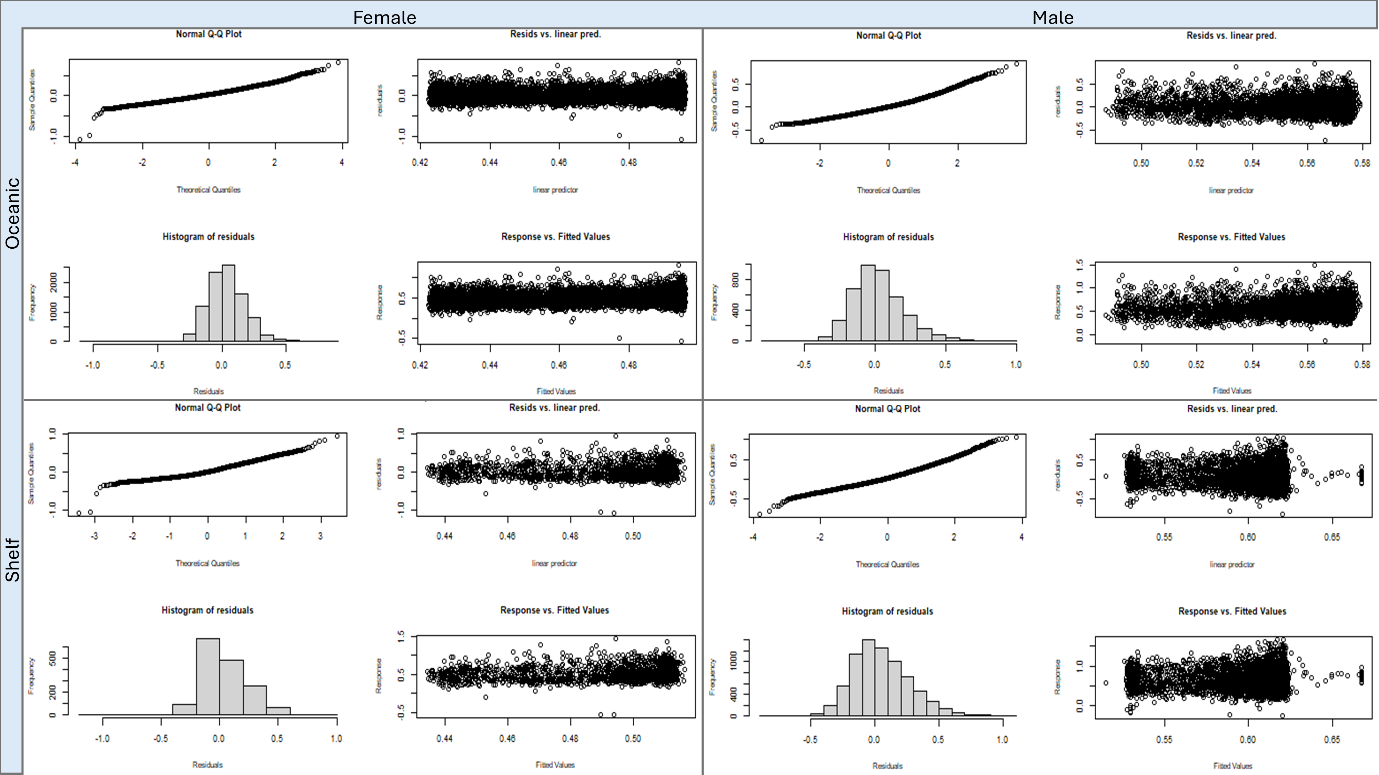


Fig. S4. Diagnostic plots from a call to “gam.check” (package mgcv; Wood 2017) for each of the GAMMs with surface residual as the response, across the 1-day temporal window. Tying in with our findings of no clear relationship between surface residual and Δ drift rate, plots of the response against fitted values indicate no clear relationship between the response and linear predictor. Model residuals however remain relatively uniformly centred around the mean.


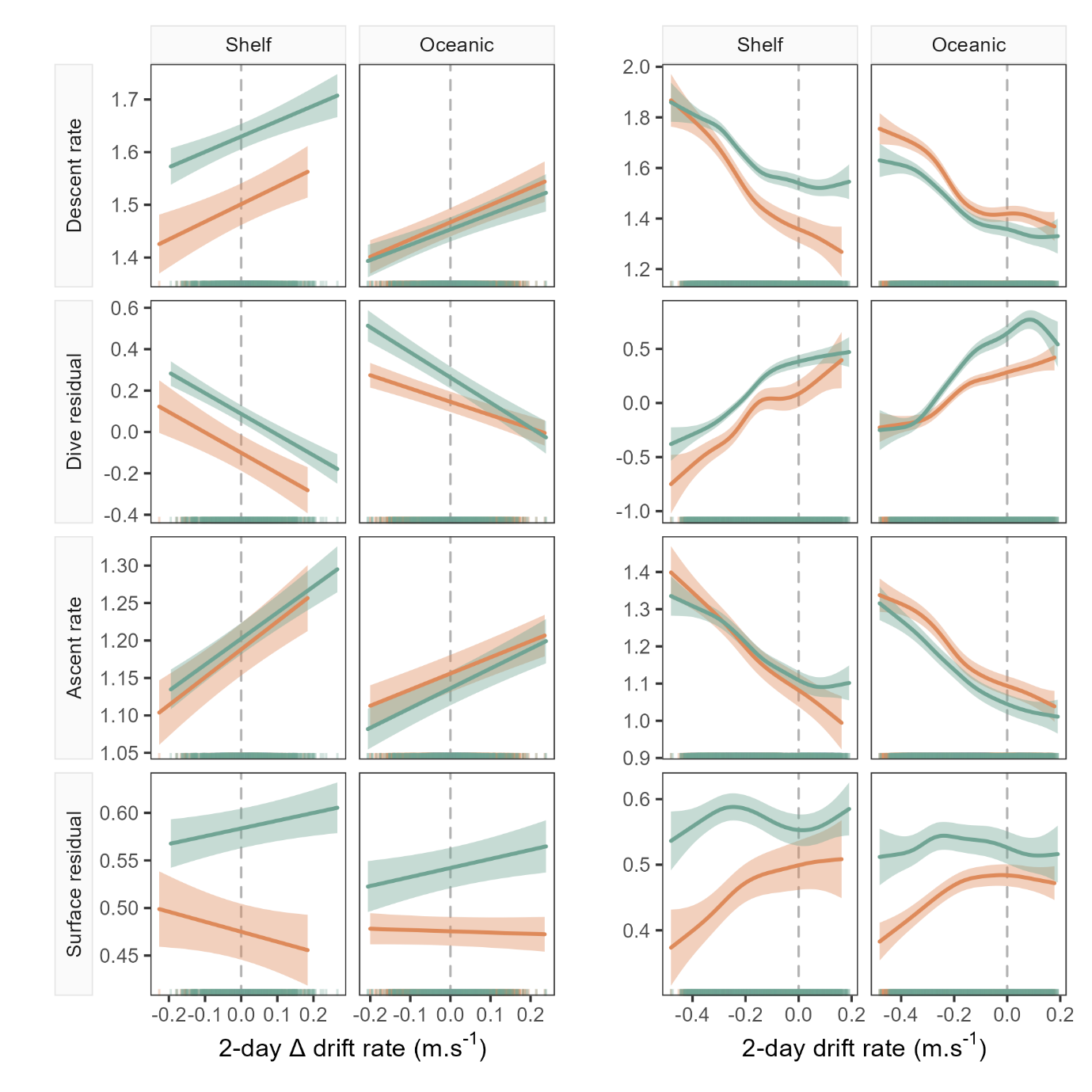


Fig. S5. Example model predictions (±95% confidence intervals) from the GAMMs fitted for the 2-day temporal window (see Figure 5 for all windows). Plots show left: the linear relationship between seal drift rate change (ΔDR) and each dive behaviour metric; and right: the partial GAMM smooths for the nonlinear relationship between daily drift rate (DR) and each dive behaviour metric.


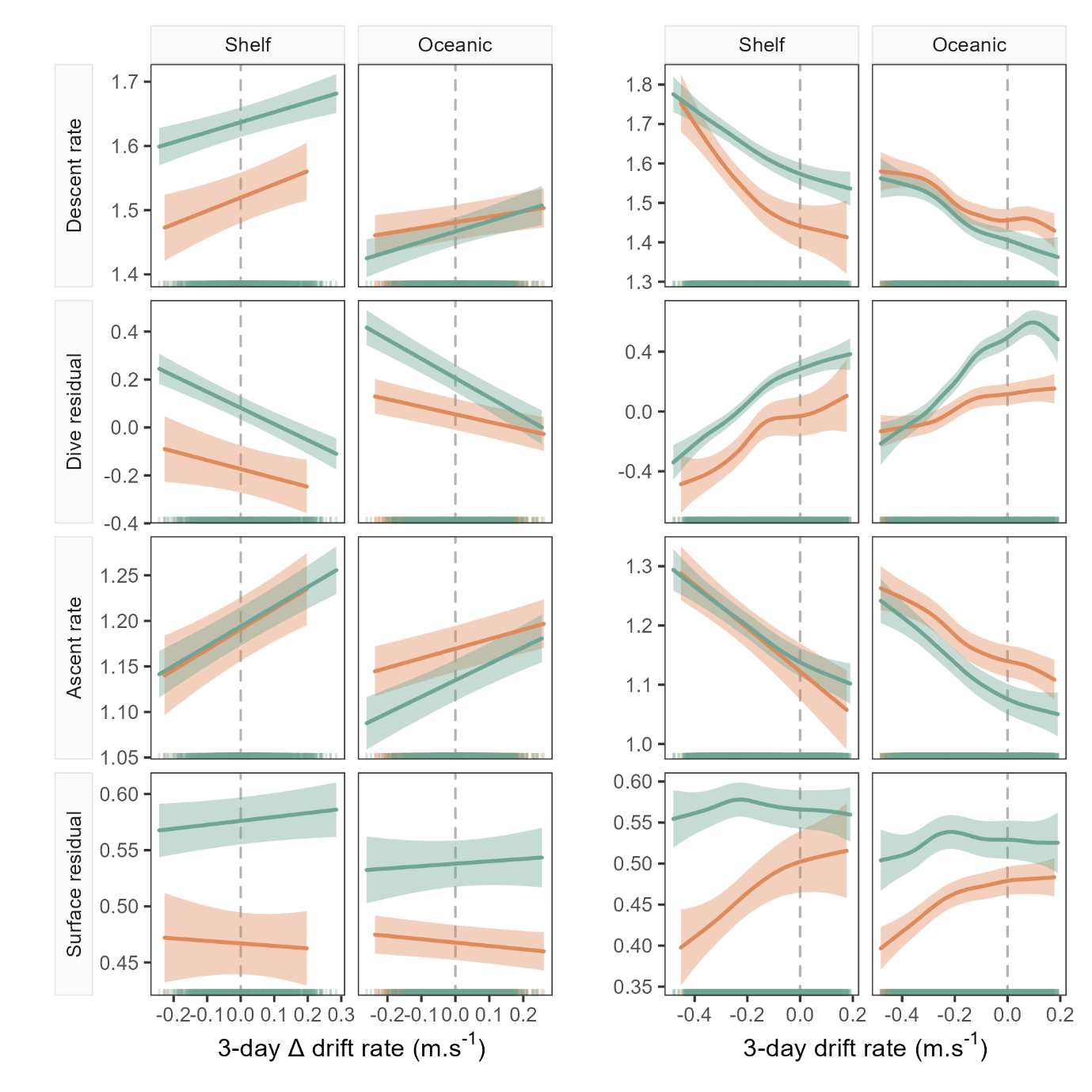


Fig. S6. Example model predictions (±95% confidence intervals) from the GAMMs fitted for the 3-day temporal window (see Figure 5 for all windows). Plots show left: the linear relationship between seal drift rate change (ΔDR) and each dive behaviour metric; and right: the partial GAMM smooths for the nonlinear relationship between daily drift rate (DR) and each dive behaviour metric.


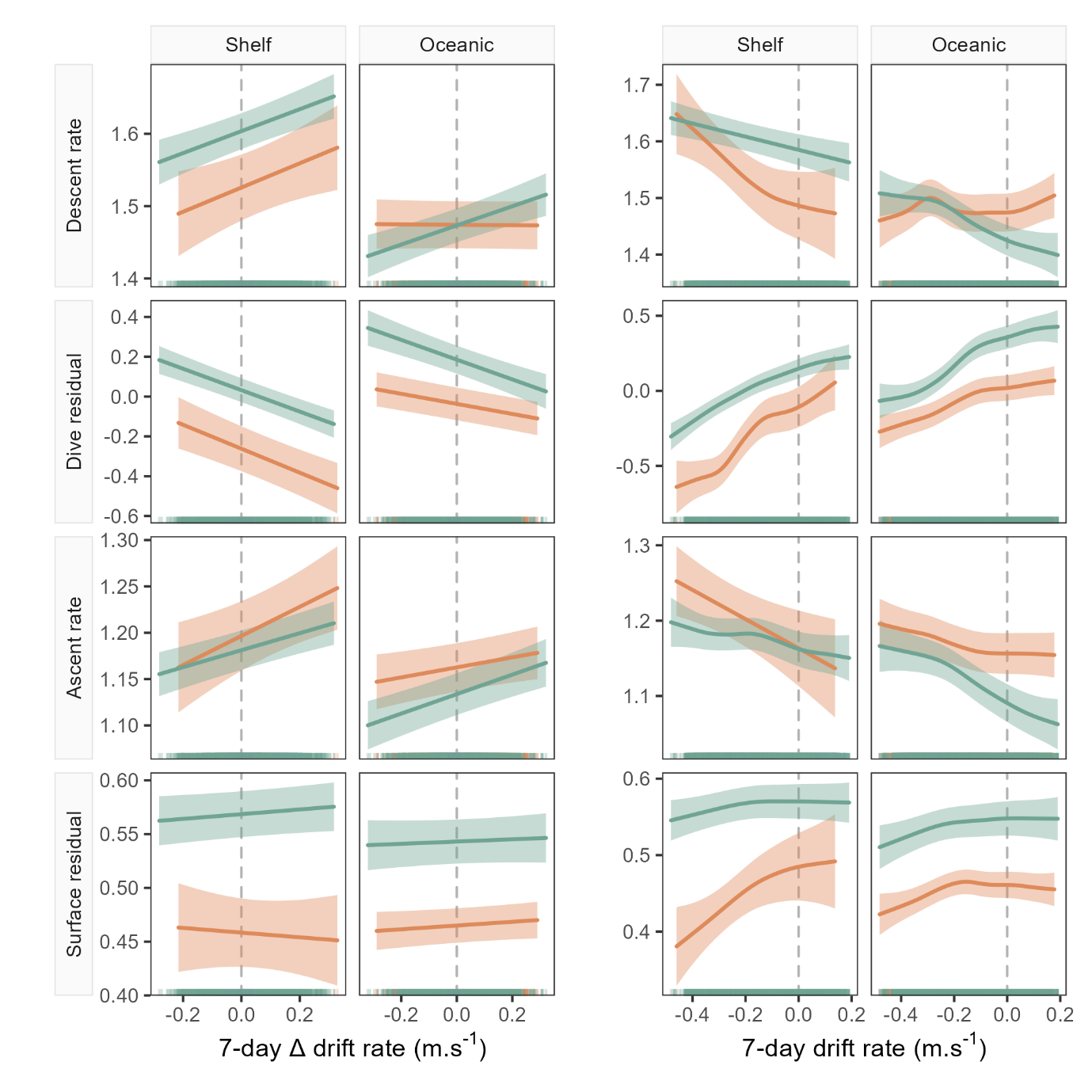


Fig. S7. Example model predictions (±95% confidence intervals) from the GAMMs fitted for the 7-day temporal window (see Figure 5 for all windows). Plots show left: the linear relationship between seal drift rate change (ΔDR) and each dive behaviour metric; and right: the partial GAMM smooths for the nonlinear relationship between daily drift rate (DR) and each dive behaviour metric.


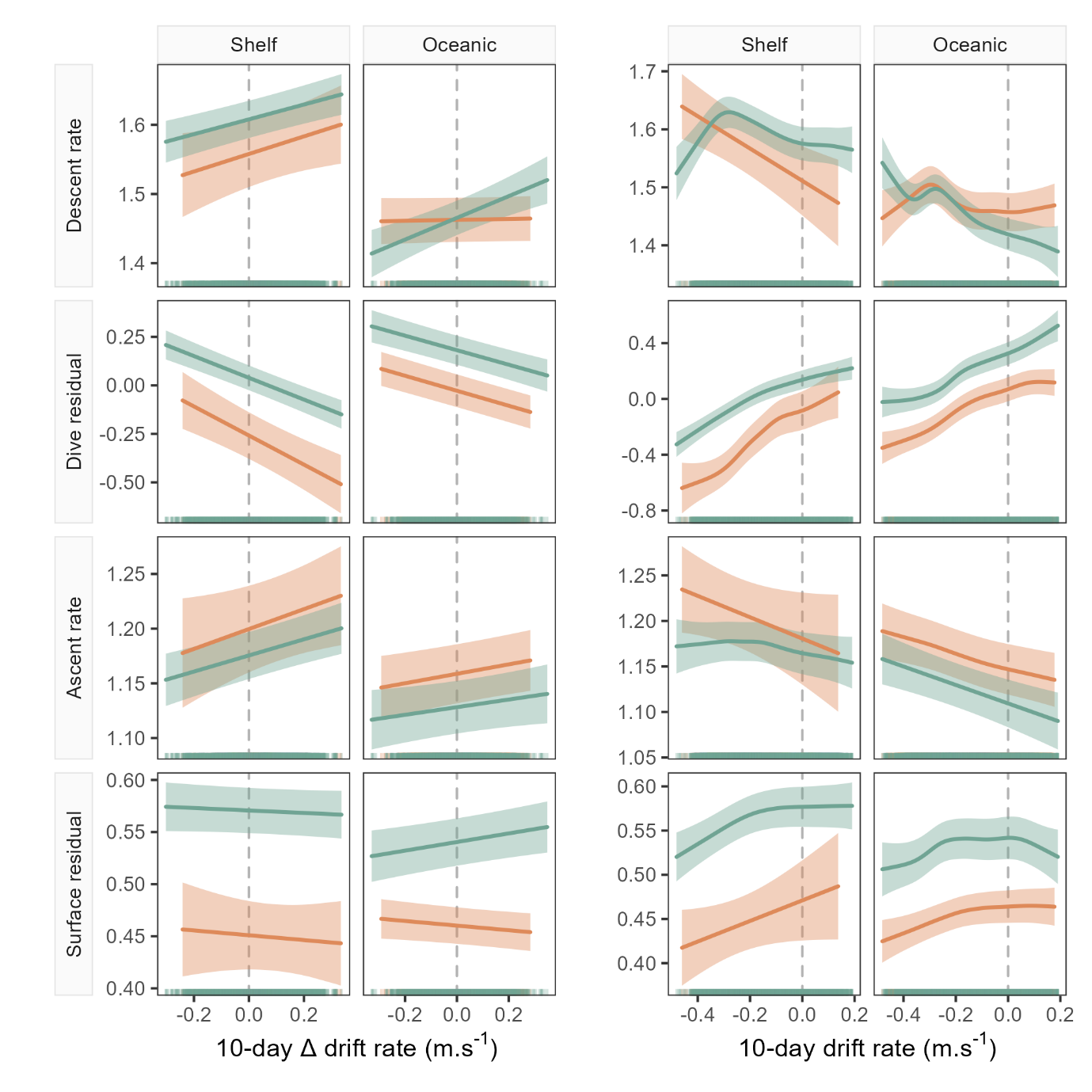


Fig. S8. Example model predictions (±95% confidence intervals) from the GAMMs fitted for the 10-day temporal window (see Figure 5 for all windows). Plots show left: the linear relationship between seal drift rate change (ΔDR) and each dive behaviour metric; and right: the partial GAMM smooths for the nonlinear relationship between daily drift rate (DR) and each dive behaviour metric.

- 1. **Modelling bottom time and hunting time responses to foraging success and buoyancy**

Following dive data preparation and filtering as outlined in Section 2.2 of the main text, we then also calculated metrics of the bottom phase of the dive, namely bottom time and hunting time. To compute bottom time we calculated the duration between the first and last dive inflection points (Allegue *et al.* 2023), denoting the bottom phase of the dive. Likewise, to compute hunting time we calculated the sum of all dive segments where the vertical speed was < 0.4m.s^-1^ (Heerah *et al.* 2015). As with the other metrics we then computed a mean of each metric across time windows spanning 1, 2, 3, 7 and 10 days. This allowed us to investigate the relationships between these metrics and foraging succes across a range of temporal scales. GAMMs were fitted using the same procedures as detailed in the main text, Section 2.4.

From outputs of the model fit, we note that the responses of both bottom time and hunting time are strongly consistent with the dive residual response to foraging success (see Table S2, and Figures S5-S9 below).

Table S2: Results from GAMM model fits not reported in the main text, between bottom time and hunting time, Δ drift rate and drift rate. “Sig.” denotes p-value significance at the following levels: “***” = p < 0.001, “**” = p < 0.01, “*” = p < 0.05, “.” = p < 0.1, “ns” = p > 0.1.

|  |  |  |  | **Dive metric linear term results** | | |  | **DR smooth results** | |  |  |
| --- | --- | --- | --- | --- | --- | --- | --- | --- | --- | --- | --- |
| **Dive metric** | **Sex** | **Habitat** | **Window (days)** | **Coeff.** | **Std. Err** | **t value** | **Sig.** | **EDF** | **F stat.** | **Sig.** | **R^2^ _adjusted_** |
| Bottom time | Female | Shelf | 1 | -18.15 | 3.39 | -5.36 | *** | 6.16 | 59.81 | *** | 0.51 |
| Bottom time | Female | Shelf | 2 | -10.11 | 1.66 | -6.08 | *** | 6.22 | 39.70 | *** | 0.42 |
| Bottom time | Female | Shelf | 3 | -5.51 | 1.44 | -3.82 | *** | 5.17 | 17.57 | *** | 0.30 |
| Bottom time | Female | Shelf | 7 | -4.23 | 0.97 | -4.35 | *** | 5.88 | 17.95 | *** | 0.27 |
| Bottom time | Female | Shelf | 10 | -5.37 | 0.78 | -6.86 | *** | 4.51 | 24.06 | *** | 0.25 |
| Bottom time | Female | Oceanic | 1 | -14.42 | 1.44 | -10.02 | *** | 7.87 | 376.01 | *** | 0.57 |
| Bottom time | Female | Oceanic | 2 | -6.19 | 0.74 | -8.40 | *** | 6.69 | 45.19 | *** | 0.32 |
| Bottom time | Female | Oceanic | 3 | -1.59 | 0.53 | -2.98 | ** | 5.68 | 9.35 | *** | 0.12 |
| Bottom time | Female | Oceanic | 7 | -1.29 | 0.30 | -4.32 | *** | 5.62 | 16.54 | *** | 0.11 |
| Bottom time | Female | Oceanic | 10 | -2.29 | 0.27 | -8.51 | *** | 1.00 | 155.24 | *** | 0.11 |
| Bottom time | Male | Shelf | 1 | -9.52 | 1.69 | -5.63 | *** | 6.40 | 109.39 | *** | 0.19 |
| Bottom time | Male | Shelf | 2 | -5.83 | 0.92 | -6.31 | *** | 6.16 | 34.50 | *** | 0.14 |
| Bottom time | Male | Shelf | 3 | -2.50 | 0.62 | -4.07 | *** | 4.35 | 17.00 | *** | 0.08 |
| Bottom time | Male | Shelf | 7 | -2.44 | 0.34 | -7.12 | *** | 4.04 | 23.56 | *** | 0.06 |
| Bottom time | Male | Shelf | 10 | -2.56 | 0.29 | -8.81 | *** | 5.29 | 26.36 | *** | 0.06 |
| Bottom time | Male | Oceanic | 1 | -13.29 | 1.80 | -7.39 | *** | 7.37 | 102.59 | *** | 0.38 |
| Bottom time | Male | Oceanic | 2 | -7.62 | 0.98 | -7.80 | *** | 6.94 | 30.89 | *** | 0.27 |
| Bottom time | Male | Oceanic | 3 | -4.04 | 0.73 | -5.55 | *** | 6.80 | 14.24 | *** | 0.17 |
| Bottom time | Male | Oceanic | 7 | -1.94 | 0.44 | -4.38 | *** | 4.37 | 15.01 | *** | 0.11 |
| Bottom time | Male | Oceanic | 10 | -2.29 | 0.39 | -5.87 | *** | 4.90 | 24.53 | *** | 0.12 |
| Hunting time | Female | Shelf | 1 | -17.51 | 3.57 | -4.90 | *** | 5.22 | 82.16 | *** | 0.52 |
| Hunting time | Female | Shelf | 2 | -11.84 | 1.67 | -7.07 | *** | 6.12 | 46.10 | *** | 0.45 |
| Hunting time | Female | Shelf | 3 | -7.51 | 1.54 | -4.87 | *** | 5.19 | 22.73 | *** | 0.36 |
| Hunting time | Female | Shelf | 7 | -4.90 | 0.99 | -4.95 | *** | 6.11 | 18.87 | *** | 0.31 |
| Hunting time | Female | Shelf | 10 | -6.01 | 0.84 | -7.20 | *** | 4.31 | 24.36 | *** | 0.28 |
| Hunting time | Female | Oceanic | 1 | -16.52 | 1.53 | -10.80 | *** | 7.75 | 349.64 | *** | 0.54 |
| Hunting time | Female | Oceanic | 2 | -8.33 | 0.77 | -10.76 | *** | 7.23 | 65.34 | *** | 0.36 |
| Hunting time | Female | Oceanic | 3 | -2.87 | 0.58 | -4.97 | *** | 5.64 | 14.74 | *** | 0.16 |
| Hunting time | Female | Oceanic | 7 | -1.85 | 0.33 | -5.64 | *** | 5.03 | 16.42 | *** | 0.11 |
| Hunting time | Female | Oceanic | 10 | -2.83 | 0.30 | -9.51 | *** | 4.09 | 45.64 | *** | 0.15 |
| Hunting time | Male | Shelf | 1 | -12.85 | 1.68 | -7.66 | *** | 7.04 | 141.49 | *** | 0.22 |
| Hunting time | Male | Shelf | 2 | -7.81 | 0.82 | -9.48 | *** | 6.13 | 60.62 | *** | 0.18 |
| Hunting time | Male | Shelf | 3 | -4.25 | 0.61 | -6.94 | *** | 4.82 | 27.23 | *** | 0.11 |
| Hunting time | Male | Shelf | 7 | -3.02 | 0.34 | -9.00 | *** | 4.69 | 27.22 | *** | 0.08 |
| Hunting time | Male | Shelf | 10 | -2.90 | 0.29 | -10.19 | *** | 5.44 | 28.19 | *** | 0.07 |
| Hunting time | Male | Oceanic | 1 | -15.87 | 2.46 | -6.44 | *** | 6.89 | 120.73 | *** | 0.38 |
| Hunting time | Male | Oceanic | 2 | -9.57 | 0.92 | -10.45 | *** | 7.62 | 49.95 | *** | 0.33 |
| Hunting time | Male | Oceanic | 3 | -5.55 | 0.73 | -7.56 | *** | 7.51 | 26.43 | *** | 0.24 |
| Hunting time | Male | Oceanic | 7 | -3.31 | 0.70 | -4.73 | *** | 4.50 | 22.21 | *** | 0.15 |
| Hunting time | Male | Oceanic | 10 | -2.48 | 0.39 | -6.39 | *** | 3.68 | 30.72 | *** | 0.13 |


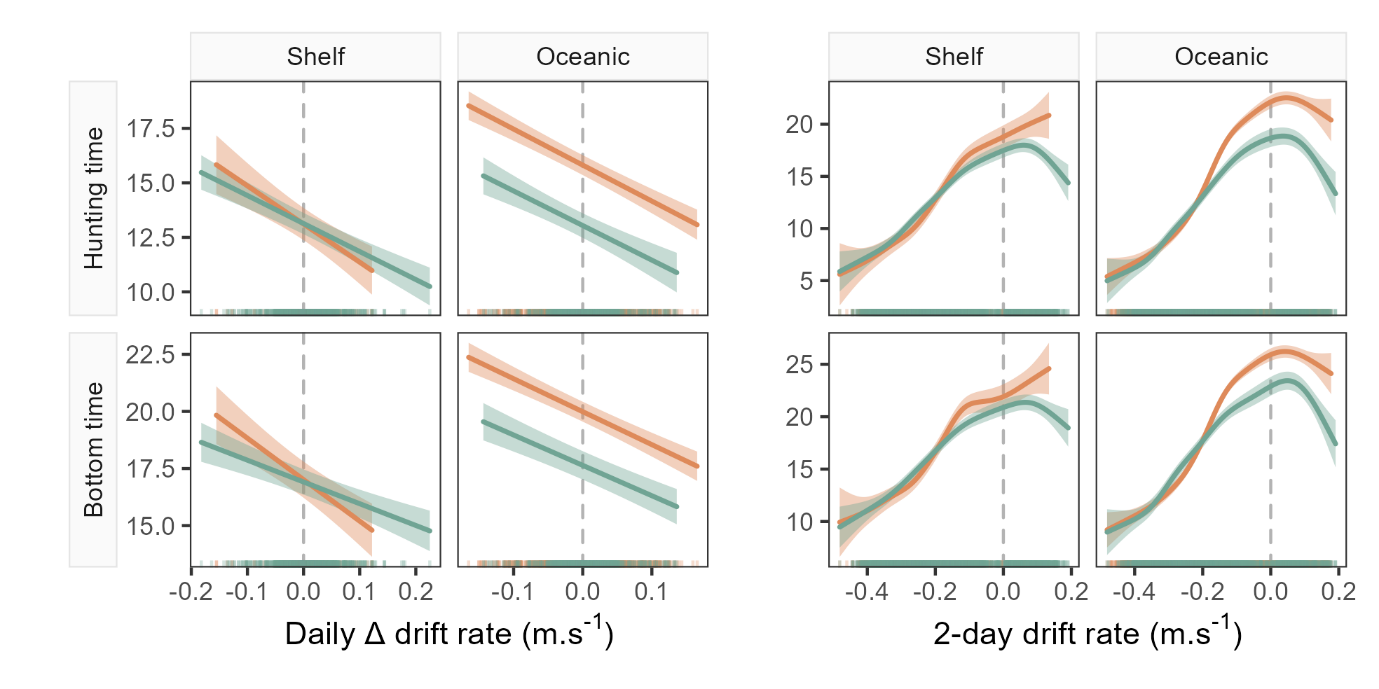


Fig. S9. Model predictions (±95% confidence intervals) from the GAMMs fitted for hunting time and bottom time across the 1-day temporal window. Plots show left: the linear relationship between seal drift rate change (ΔDR) and each dive behaviour metric; and right: the partial GAMM smooths for the nonlinear relationship between daily drift rate (DR) and each dive behaviour metric. Note, in all cases the sign of the linear slope coefficient is consistent with relationships observed for the dive residual.


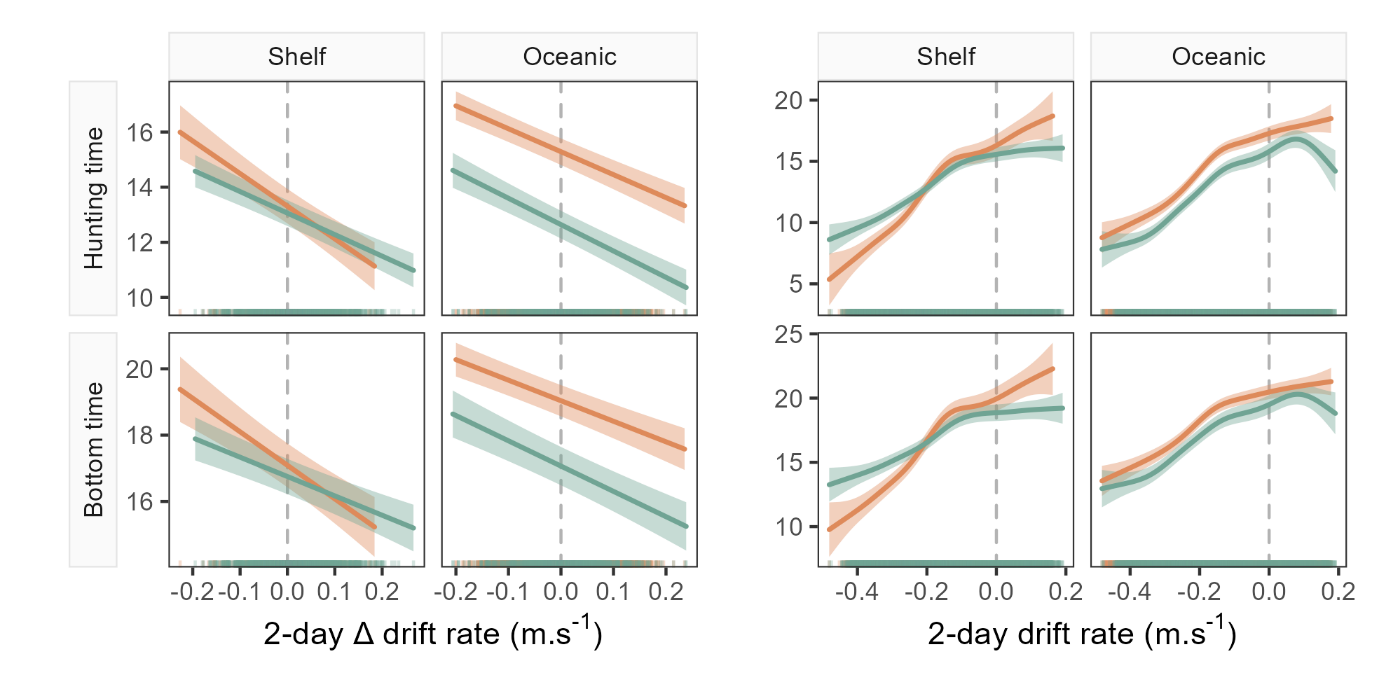


Fig. S10. Model predictions (±95% confidence intervals) from the GAMMs fitted for hunting time and bottom time across the 2-day temporal window. Plots show left: the linear relationship between seal drift rate change (ΔDR) and each dive behaviour metric; and right: the partial GAMM smooths for the nonlinear relationship between daily drift rate (DR) and each dive behaviour metric. Note, in all cases the sign of the linear slope coefficient is consistent with relationships observed for the dive residual.


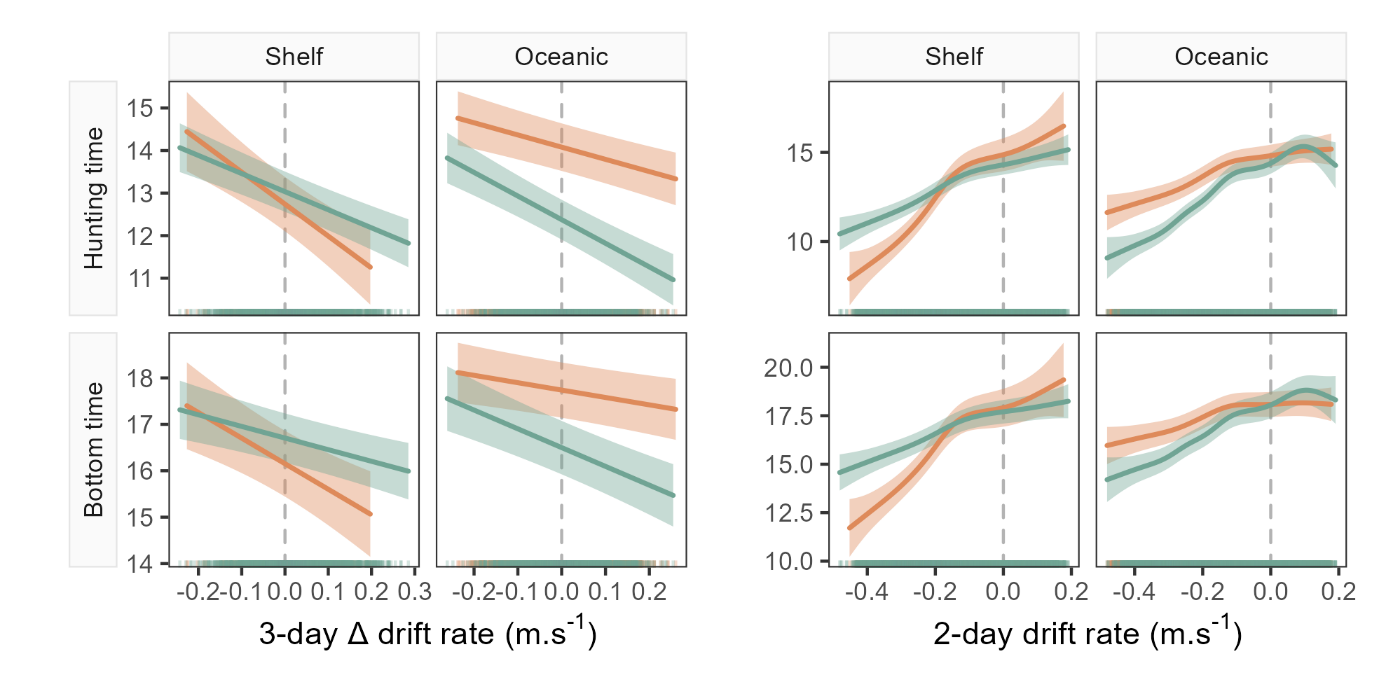


Fig. S11. Model predictions (±95% confidence intervals) from the GAMMs fitted for hunting time and bottom time across the 3-day temporal window. Plots show left: the linear relationship between seal drift rate change (ΔDR) and each dive behaviour metric; and right: the partial GAMM smooths for the nonlinear relationship between daily drift rate (DR) and each dive behaviour metric. Note, in all cases the sign of the linear slope coefficient is consistent with relationships observed for the dive residual.

**
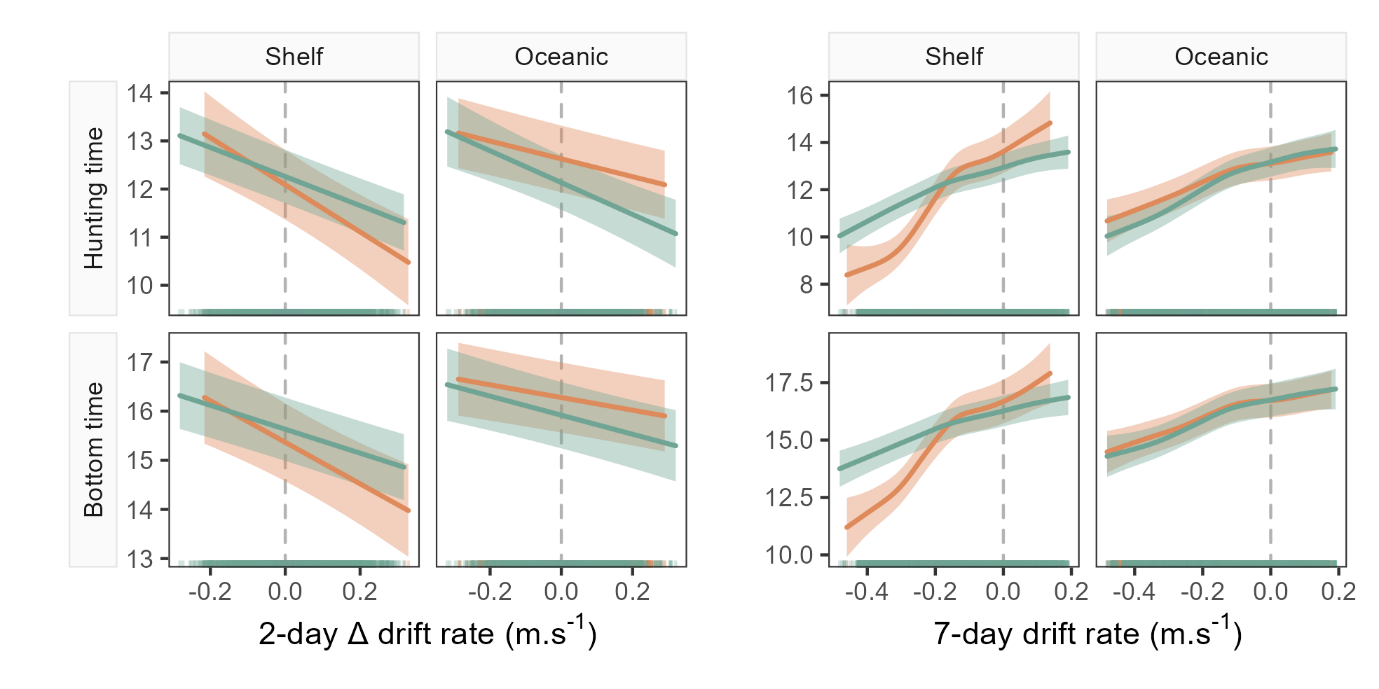
**

Fig. S12. Model predictions (±95% confidence intervals) from the GAMMs fitted for hunting time and bottom time across the 7-day temporal window. Plots show left: the linear relationship between seal drift rate change (ΔDR) and each dive behaviour metric; and right: the partial GAMM smooths for the nonlinear relationship between daily drift rate (DR) and each dive behaviour metric. Note, in all cases the sign of the linear slope coefficient is consistent with relationships observed for the dive residual.

**
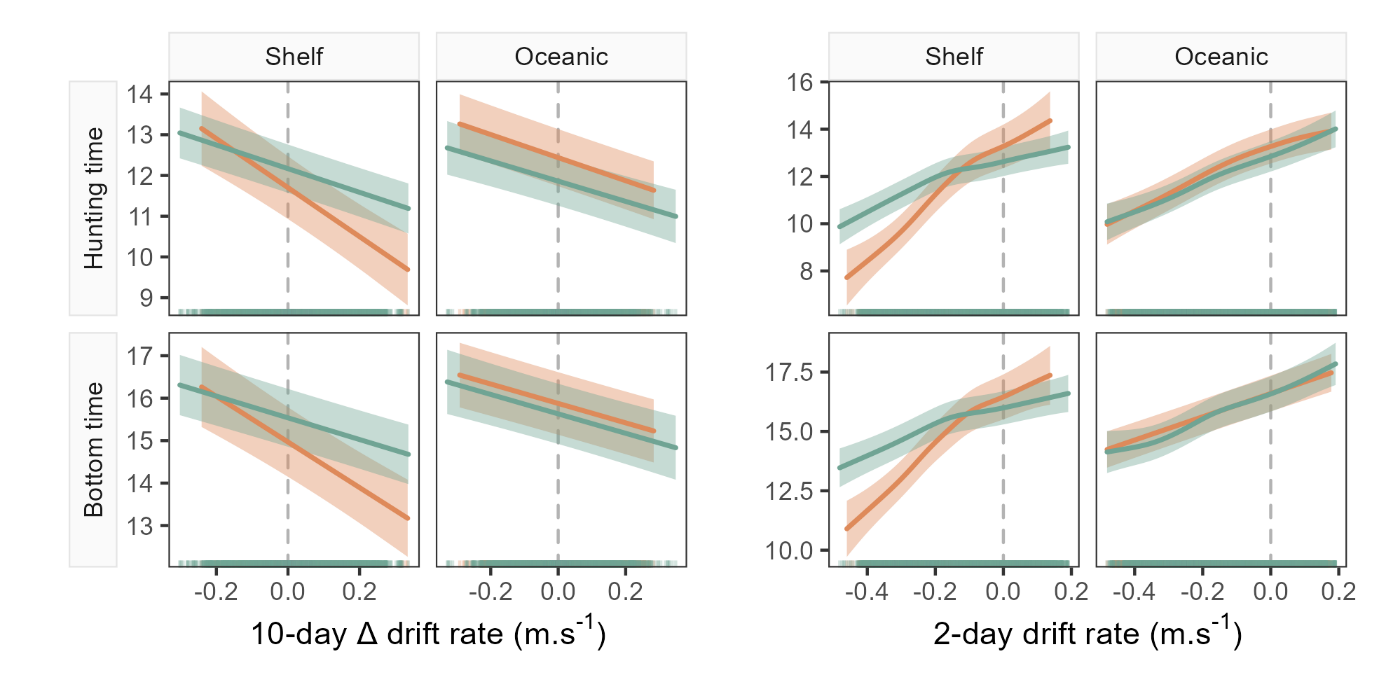
**

Fig. S13. Model predictions (±95% confidence intervals) from the GAMMs fitted for hunting time and bottom time across the 10-day temporal window. Plots show left: the linear relationship between seal drift rate change (ΔDR) and each dive behaviour metric; and right: the partial GAMM smooths for the nonlinear relationship between daily drift rate (DR) and each dive behaviour metric. Note, in all cases the sign of the linear slope coefficient is consistent with relationships observed for the dive residual.

**References**

1.

Allegue, H., Réale, D., Picard, B. & Guinet, C. (2023). Track and dive-based movement metrics do not predict the number of prey encountered by a marine predator. *Movement Ecology*, 11, 1-19.

2.

Heerah, K., Hindell, M., Guinet, C. & Charrassin, J.-B. (2015). From high-resolution to low-resolution dive datasets: a new index to quantify the foraging effort of marine predators. *Animal Biotelemetry*, 3, doi 10.1186/s40317-40015-40074-40313.

3.

Wood, S.N. (2017). *Generalized additive models: an introduction with R*. CRC press.
